# Parents’ views on child physical activity and their implications for physical activity parenting interventions: a qualitative study

**DOI:** 10.1186/1471-2431-12-180

**Published:** 2012-11-20

**Authors:** Georgina F Bentley, Joanna K Goodred, Russell Jago, Simon J Sebire, Patricia J Lucas, Kenneth R Fox, Sarah Stewart-Brown, Katrina M Turner

**Affiliations:** 1 Centre for Exercise, Nutrition & Health Sciences, School for Policy Studies, University of Bristol, Bristol, UK; 2 Centre for Research in Health and Social Care, School for Policy Studies, University of Bristol, Bristol, UK; 3 Warwick Medical School, University of Warwick, Coventry, UK; 4 School of Social and Community Medicine, University of Bristol, Bristol, UK

**Keywords:** Physical activity, Parenting, Intervention

## Abstract

**Background:**

Establishing healthy physical activity (PA) behaviours in early childhood is important for future PA behaviours. Parents play a central role in young children’s PA. However, there is currently little research on parenting interventions to increase child PA. This study was formative work to inform the content of a pilot randomised-controlled trial.

**Methods:**

In-depth telephone interviews were carried out with 32 parents of 6 to 8 year old children residing in two areas that varied in their socio-economic characteristics, in Bristol, UK. Data were analysed thematically using a framework approach.

**Results:**

Most parents described their child as being active or very active and indicated that they did not perceive a need for an increase in their child’s PA. Parents used a variety of visual cues to make this judgement, the most common being that they perceived their child as having lots of energy or that they did not view them as overweight. Parents reported environmental factors such as monetary cost, time constraints, lack of activity provision and poor weather as the main barriers to their child’s PA. Parental support and child’s enjoyment of PA appeared to be important facilitators to children participating in PA.

**Conclusion:**

Improving parents’ knowledge of the PA recommendations for children, and increasing their awareness of the benefits of PA beyond weight status may be an important first step for a parenting PA intervention. Although parents commonly perceive environmental factors as the main barriers to their child’s PA, parental concern about low levels of child PA, their capacity to support behaviour change, child motivation, self confidence and independence may be key areas to address within an intervention to increase child PA. Effective methods of helping parents address the latter have been developed in the context of generic parenting programmes.

## Background

The benefits of physical activity (PA) in adults have been well documented and include decreased risk of obesity, type-2-diabetes and all cause mortality [1-3]. Among children, PA has been associated with improved mental well-being [4] and lower levels of cardiovascular disease risk-factors [5,6]. Research suggests that children do not engage in the recommended amount of 60 minutes moderate-to-vigorous PA per day [7,8]. The early primary school years (6–8 years) is a critical time for physical skill development [9,10] and is the period in which PA behaviour is formed [11]. There is evidence that PA behaviours in childhood track into adulthood [12,13] and therefore establishing healthy behaviours in early childhood is important for the current and future health of children.

Increasing children’s PA levels is a key UK government public health policy target [14] but presents a major challenge for public health practitioners and promoters. The majority of interventions to increase PA in children are school-based [15-17] and reviews highlight that many school-based interventions have had limited success [16,18].

Parents are able to influence their children’s behaviour and play an important role in encouraging children to be physically active [19]. Research has shown that parental support (i.e., parental self-efficacy associated with child PA, parents’ PA behaviour, and parents’ perceptions of the value of PA) [15,20,21] and logistic support (i.e., providing equipment for PA and taxiing children to activities) [22-24] are important correlates of PA levels in children. Parenting programmes have shown to be effective in preventing childhood obesity [25-27] but parenting interventions to promote children’s PA is an understudied area of research. There may be particular parenting skills which would encourage increased PA in children and working with parents is a potential intervention route.

Parents’ poor recognition of children’s PA levels could present an important barrier to behaviour change [28,29]. However, we do not currently know how parents’ assess their child’s PA. This may be particularly important for interventions as parents with inactive children have been reported to overestimate their child’s PA [30]. In addition, parents’ perceived barriers to PA have been consistently negatively associated with child PA [20] and may be important issues to address within the content of an intervention. Reported parental barriers have included lack of time [31,32], monetary cost [32,33], lack of opportunities to be active [33], the local environment, safety concerns, increasing distances between homes and school, home distractions and peer pressure for sedentary activities (e.g. screen-viewing) [31].

The UK’s Medical Research Council provides a development-evaluation-implementation model to guide the development and testing of complex interventions [34]. A key element of this process is the developmental stage in which intervention elements are developed based on existing evidence and pilot research. The present study is situated at the developmental stage and the information arising from it will be used to inform the content of a pilot randomised-controlled trial consisting of a parenting intervention aimed at increasing the PA among 6–8 year old children. Interviews were held with parents to examine factors that may influence their child’s PA levels, specifically: a) parents’ awareness of their 6–8 year old child’s PA; b) the parents’ perceived barriers to their 6–8 year old child’s PA.

## Methods

Interviews were held between January and March 2011 with parents of children aged 6 to 8 years old. The study was approved by the School for Policy Studies ethics committee at the University of Bristol and written informed consent was obtained for all participants.

### Participants

Parents were recruited from a low social economic status (SES) area and a mid-SES area in Bristol, UK. Recruitment was carried out via two methods: 1) letters describing the study and inviting parents to take part in an interview were sent home with the children in Years 2 and 3 at two schools in each of the study areas; and 2) face-to-face contact with parents at community activities (including children’s centres, after school clubs and church clubs), and at the school gates at child pick-up time. Each parent was provided with a study information sheet. Parents that agreed to take part in the study gave written consent. Parents were eligible to take part if they were the main carer for a 6 to 8 year old child and lived in either of the two research locations. Fifty-seven parents in total were recruited and consented to take part in an interview (46% from the low-SES area and 54% from the mid-SES area). Parents were interviewed on the basis of who could be contacted first whilst attempting to get an equal number of parents in each of the two study areas. Data collection ended when data saturation had been reached, (i.e., no new themes were emerging from the analysis).

### Data collection

A semi-structured interview guide was used. The guide focused on questions relating to children’s PA levels and barriers to participation in PA, e.g. questions were asked about parents’ opinions of their child’s PA levels, how they gauge their child’s PA levels, barriers to their child’s PA, and support for their child’s PA. Interviews were arranged and conducted by GFB and JKG and lasted between 10 and 47 minutes (mean = 22 minutes). The interviews were conducted by telephone because it is a convenient method for parents [35,36] and studies have shown that parents are more likely to answer potentially sensitive questions over the telephone [37]. The interviews were audio recorded and the two interviewers listened to each other’s recordings to ensure consistency in interviewing. Interviews were transcribed verbatim and anonymised. GFB and JKG listened to the recordings to check for accuracy with the transcriptions and any differences were reconciled. Data collection and analysis proceeded in parallel.

### Analysis

The data were analysed thematically [38]. The approach involved researchers reading and re-reading the interview transcripts in order to identify emerging themes and to develop a coding frame. Whilst all data were read and considered, the main aim of the analysis was to identify factors that would need to be taken into account when developing a parenting programme. The coding frame was provisionally tested with a sample of three transcripts by three members of the research team. Discrepancies were discussed and the coding frame was refined.

Once the coding frame was finalised, transcripts were imported into NVivo (Version 9.0, QSR, Southport, UK) to allow for electronic coding and retrieval of data. To assist with the systematic interpretation of the data, a framework approach [39] was used, which allowed for *a priori* as well as emergent themes. Using headings from the thematic coding frame a chart of the data was created, with each participant represented across all themes. This enabled comparisons to be made within and across the interviews, identification of deviant cases, and the views parents held towards a particular issue to be highlighted. Quotes reproduced in this paper have been labelled with information indicating whether the interviewee was a mother or father, and their area of residence (low-SES or mid-SES).

## Results

Thirty-two parents were interviewed, 15 (47%) from the low-SES area and 17 (53%) from the mid-SES area (Table 1). The majority of parents interviewed were female (90%) and most of the children within the 6 to 8 year age criteria were also female (62%). Six (40%) parents from the low-SES area and one (6%) of the parents from mid-SES were lone parents.

**Table 1:** **Demographic characteristics of parents** (**n** = **32**)

	**Mean**	**Range**
**Age** (**years**)	38.2	25.5 – 48.0
Low-SES	35.8	25.5 – 47.1
Mid-SES	40.3	26.8 – 48.0
**Number of children in household**	2.2	1-4
Low-SES	2.1	1-4
Mid-SES	2.2	1-4
	**N**	**%**
**Gender** (**female**)	29	90.6
Low-SES	15	100
Mid-SES	14	82.3
**Lone parent**	7	21.9
Low-SES	6	40.0
Mid-SES	1	5.9

SES = social economic status.

This paper focuses on factors that were identified during the analysis that would need to be considered when developing a parenting intervention to increase children’s PA. These included parents’ awareness of their child’s PA levels, barriers to their child’s PA, and parental support for their child’s PA.

### Parents’ awareness of their child’s PA levels

The majority of the parents felt that their child was either active or very active. Some parents felt that their child was fairly active but had room for improvement, the majority of these respondents were from the mid-SES area. Only a small number of parents reported that their child had low levels of PA.

A couple of parents from the mid-SES area indicated that it was difficult to judge whether children were getting enough PA. One parent showed some dissonance between believing that her child is active enough and being uncertain that they actually are.

“I know that [Child’s name] gets enough but you know, do I really know?” (Mother, Mid SES)

Another parent highlighted their understanding of a desirable amount of PA for children yet she was uncertain of the recommendations.

“I can’t quantify very well how much exercise my children get… and what would the desirable, I have no idea about whether they’re doing (enough)… so I’d be really interested in knowing what, where we’re at on that kind of thing” (Mother, Mid SES)

Parents gauged their child’s activity levels by using a combination of visible cues. For instance, they frequently suggested that their child was active because they perceived them to have high levels of energy or always being ‘on the go’.

“Very, very, very active…at home he can’t sit still, he doesn’t sit still” (Mother, Low SES)

“She’s on the go all the time, doesn’t sit down for long. Got loads of energy.” (Mother, Low SES)

Many parents placed emphasis on PA’s role in weight management and associated their child’s activity levels with their weight or size. An active child was equated with them being slim and an inactive child was equated with having excess weight.

“Yeah, she’s quite slim as well…You can tell she, she can fit into 5 to 6 year old skirts, so she’s quite a slim child…Oh yeah she burns it as quick as she eats it.” (Mother, Mid SES)

“He doesn’t do much of anything and he’s put on quite a bit of weight.” (Mother, Low SES)

A small number of parents reported their child was sufficiently active because their fitness levels were good, and felt that if they were physically fit, they were active enough.

“…he is very strong, and his ability to walk for miles on end is quite remarkable.” (Mother, Mid SES)

Children’s interest or enjoyment of PA was also linked to them being active.

“Oh he’s very active, his favourite lesson in school is PE, so yeah he likes to be active.” (Mother, Low SES)

Some parents who described their child as active felt that their child might need their activity to be moderated.

“…so it’s not as if it’s a case of me for instance, “well let’s do this, let’s do that”, it’s the other way round, and it’s trying to maybe sometimes reign her in.” (Mother, Low SES)

Screen-viewing was seen as an indicator for low levels of PA, and parents gave descriptions of the child’s screen-viewing behaviours as well as their preference for it.

“Well, if you said to [child’s name], “do you want to go and play out at the park, or do you want to go on your Xbox?” he will play on the Xbox.” (Mother, Low SES)

“She is at home a lot and all she’s into just watching DVDs, she’s really into princesses and fairies and … you know.” (Mother, Low SES)

### Parents’ perceived barriers and facilitators to their child’s PA

The child’s personality and enjoyment of PA appeared to influence how active their parent perceived them to be. For instance, some parents found that their child’s preferences made it difficult to encourage activity.

“That’s pretty much all he likes. He doesn’t like football. None, none of mine are into football, they prefer the computer.” (Mother, Low SES)

“… once he’s up and doing it he’s alright, but like walking and things like that he moans all the time.” (Mother, Mid SES)

Whereas others found that their child naturally wants to be active.

“She likes being out in the garden or she wants to go to the park and if we go to the park she’ll be on her scooter.” (Mother, Mid SES)

On many occasions parents reported their child’s enjoyment and enthusiasm for a particular organised PA led the parent to choose that activity for their child. However, in other instances barriers prevented the child from engaging in their preferred PA. For instance, the cost of organised activities and a lack of provision of a specific organised activity proved to be a barrier.

“He always want to play football in a club. He ask me, he keep asking me every, every time and I say I can’t afford.” (Father, Mid SES)

“She used to do dance classes, but they don’t seem to do that at the Juniors.” (Mother, Low SES)

When talking about how to increase children’s PA, many parents instinctively talked about increasing organised activities for their child and how additional activities would be problematic because of the parents’ work, existing commitments for parents and the child, and shortage of time.

“Yeah, erm just the start time and if I’m at work …because I, I work three days a week until six in the evening …because some things just start too early and I don’t like having to ask other people to take her.” (Mother, Low SES)

“Er, erm, she’s mentioned dancing but to be quite honest … we can’t really fit it in.” (Mother, Mid SES)

Managing several children, especially of different ages, was also mentioned as a difficulty by some parents, in that preparing to go out for the day is difficult, that different age stages made it difficult to do things as a family, and siblings’ organised activities made it difficult to do things as a whole family.

“I might think in the morning, right we're going to do so, and so, and so and so after school today and then it gets to that point and someone says, oh yeah well I've got to go wee and then you’ve got them going in and back and forth and the whole plan just goes out the window.” (Mother, Low SES)

“… making a two year old run here, there and everywhere after her older sister all the time is… a real genuine tension that gets worse as the younger one grows up…” (Mother, Mid SES)

Some parents described barriers to their child’s participation in PA that related to their own priorities or capacity to facilitate PA which related to their circumstances.

“He wanted to do and he was meant to [play rugby]… it wasn’t within our area, erm, and a friend … he was going to go with a friend. We couldn’t sort out the transport, and you know the kids talk amongst theirselves, they kind of arranged it and it couldn’t come off with us adults I’m afraid.” (Mother, Low SES)

“Well she likes going to the park, but again it will more or likely probably we have to do it when I’m really tired, because I’m the only parent here, so that again sometimes impedes you know.” (Mother, Low SES)

Other barriers that parents said prevented their child’s PA included lack of safe outdoor space, transport, location of activity, and bad weather.

“Yeah and she used to do swimming after school as well but then it goes to winter time and getting the bus back from the swimming baths at 5 on a night when they’re soaking wet isn’t very good either.” (Mother, Low SES)

“Well we bought a trampoline back in the summer and he does love that, but of course it’s been out of bounds with it being so wet and cold.” (Mother, Low SES)

“You know, is just, you know, because we live in an area there’s always car, you know parking, is not safe, you know to even play football.” (Father, Mid SES)

Parents also suggested potential solutions to some of these barriers. For instance, some parents’ mentioned that PA doesn’t necessarily have to bear a financial cost.

“Just go out and discover what is around you. I think a lot of mums think we have got to spend money but you don’t you know… I would probably recommend going to some lovely local parks where it cost you no money.” (Mother, Mid SES)

Many parents utilised after school activity provision and one parent highlighted that perhaps children mind less about bad weather than parents.

“I mean it doesn’t hurt the kids to at a park running around if you’re just sat there watching them… you know they don’t mind and they don’t care what weather it is, they really don’t care [laughs].” (Mother, Low SES)

While juggling the needs of siblings was reported as making some activities problematic, peer support for children was reported to be an important facilitator for activity. Some parents reported that having either a sibling or friend attending an activity was a positive reason for their child attending the same activity.

“…the thing is of course having a big sister they always want to do what the older one does don’t they?” (Mother, Mid SES)

“It’s the peer thing even at that age it’s a big issue, you know. If you’ve got a couple of friends going then you’re more likely to get them…” (Mother, Mid SES)

### Parental support of their child’s PA

Parents from the low-SES area often reported providing support and encouragement for unorganised activity and active play, whereas parents from the mid-SES area more commonly reported providing support for organised activities and groups. It became evident that parents’ felt that for their child, parental support and staying close to their mother was important.

“The only thing is, erm, is getting my children to take part in after school activities is more difficult because they like coming home.” (Mother, Mid SES)

“I think … if it was something that we did together, erm, I think I’d do okay, but he lacks confidence to … do something independently from me.” (Mother, Low SES)

“She’s quite shy, when she first starts new things, I think if I weren’t to stay with her the first couple of times then she probably wouldn’t want to do it.” (Mother, Low SES)

Parents commonly reported providing logistic support to facilitate their child’s PA, such as encouraging a particular PA and providing PA equipment (including parents from the low-SES area who had reported that a lack of money was a barrier). However, this external motivation appeared to have limited success in encouraging the targeted PA. These parents tended not to talk about their child’s own motivation or preference for PA in the interview.

“I did get him to think about joining the football club but that just quite didn't happen for some reason but I can't remember why.” (Mother, Low SES)

“No she’s quite lazy actually, I’ve got to sort of push her to even go out on the trampoline in the garden and even go on the Wii fit. You know she’s quite happy doing nothing… I mean we bought her a bike last year, and she didn’t even get on it and ride it, she just hasn’t got any interest in things like that, so it is quite hard.” (Mother, Low SES)

There was an acknowledgment by some parents of the importance of child-led play and supporting a child’s motivation to be active.

“I like my two to be able to play without being sort of like guided on how to do it.” (Mother, Mid SES)

“Well I guess you’ve got to offer them things that they’re interested in.” (Mother, Low SES)

Table 2 summarises the key findings of this paper and the implications for a PA/SV parenting programme.

**Table 2:** Implications of results for parenting interventions to increase PA in children

**Key components to be addressed within a parenting PA intervention**	**Desired outcome**	**Intervention content to achieve outcome**
Improving parents’ awareness of child’s PA levels	• Improving understanding of the PA recommendations	• PA recommendations for children
		• What are the benefits of PA?
	• Increasing parents’ perceived need for more PA	• What is PA and what counts?
		• Tools to monitor PA
• Overcoming barriers to PA and increasing opportunities to be active	• Providing parents with ideas and skills for motivating their children to engage in PA	• Providing local activity directory
	• Trouble-shooting common barriers such as cost/lack of provision/weather	• Supporting active travel and lifestyle activity
	• Providing parents with skills to deal with conflict around PA to avoid negative associations with PA	• Generating ideas for low-cost/free, and wet weather games and activities
		• Discussion around how modern living and today’s culture affects PA and sedentary behaviour
		• Balancing boundaries and autonomy with risk and safety
		• Scheduling and planning activities
		• Managing conflict around PA (parenting skills including giving choice, setting boundaries, positive discipline and praise)
Helping parents’ to support children’s intrinsic motivation to be active	• Supporting the development of children’s physical skills and confidence to be active	• How parents can practically support their child’s PA development (e.g. game modification)
	• Developing children’s self confidence and independence	• Importance of physical skills development
	• Supporting parents’ confidence and competence in supporting their child’s PA	• Developmental stages for physical skills for children and having appropriate expectations
	• Encouraging activity and supporting fun and enjoyment	• Using praise and positive discipline
		• How can parents emotionally support their child’s PA confidence, independence and autonomy)
		• Making activities fun
		• Generating ideas for family activities

## Discussion

This study explored the views of parents of children aged 6–8 years pertaining to their child’s PA and the factors that may facilitate and hinder PA for their child. The interviews indicate a variety of factors that may influence a child’s activity levels, which are important to take into consideration when designing the content of an intervention to increase PA levels in children. The data presented in Table 2 provide a summary of how the key issues that were raised in this study could be addressed in the development of a PA parenting programme. These issues are discussed in detail below.

### Improving parents’ awareness of their child’s PA levels

Most parents considered their child to be active or very active, and they used a variety of visual cues to make this assessment. Most prominently, a child that displayed a lot of energy was seen as an active child. However, there is evidence that parents of inactive children overestimate their child’s PA [30]. As the cultural norm has shifted to support a more sedentary lifestyle there may be a loss of intuitive knowledge of the value of PA. The normal desire for children to move about and explore their surroundings may seem to parents to be inappropriate and in need of correction. Suppression of such a normal desire could lead children to appear to be hyperactive.

Weight status was also commonly used by parents as an indicator of sufficient child PA. However, it has previously been shown that parents do not have accurate perceptions of their child’s weight [40] and they may look for ways to validate their child’s size as being 'normal' whether over or underweight, and define their comparators (e.g., other children, family members, clothes size) in ways that achieve this [41]. The use of visual cues may lead to an inaccurate perception of their child’s PA levels, and therefore parents may not consider an increase of PA for their child as necessary. Indeed, only a few parents in this study commented that their child would benefit from increasing their PA. These findings align with previous observations that parents who believed their child to be sufficiently active did not promote PA for their child [42]. Thus, improving PA awareness is an important first step for interventions aiming to increase PA [43,44]. This may be achieved by facilitating realistic perceptions through knowledge and tools to monitor PA and studies have suggested that self monitoring [45] and personalised feedback [44] may be effective tools to help promote PA awareness.

Parents may be unaware of PA recommendations for children [46]. In this study it appeared that parents were unaware of the UK PA recommendations. Parents did not use time references to reinforce their judgement of their child being sufficiently active but seemed to place emphasis on the role of PA in preventing overweight. Educating parents about the current PA guidelines for children and how children can meet these recommendations should form a significant component in a parenting PA intervention. In addition, promoting the benefits of PA beyond weight control is important [43,45], such as promoting the short term and long term benefits [44] and facilitating an awareness of the relationship between weight status and PA [43]. Alongside these suggestions for increasing parents’ awareness of their children’s PA levels lies the possible relevance and importance of increasing awareness of recommendations for adult activity levels and encouraging more PA amongst parents themselves. If parents identify with the benefits of PA for themselves they are likely to want to enable this for their children. Such motivation would be more authentic than motivation based on externally derived recommendations.

### Overcoming barriers to PA and increasing opportunities to be active

The key environmental barriers reported by parents related to cost, time, lack of safe outdoor space, lack of activity provision, weather, and transport. It is not possible to modify these barriers in the context of a parenting intervention, nevertheless they should be addressed and tools for overcoming or reducing these barriers discussed alongside parenting interventions.

Inclusion of information and support for low-cost activities and active play is likely to be important for parenting PA interventions to address [40] and the promotion of low–cost activities such as active travel and active play has been identified as a useful strategy to increase family activity in lower socioeconomic areas [19]. In addition, parents cite lack of safe outdoor play space as a barrier to their child’s PA and therefore interventions should consider facilitating strategies to increase perceived safety [47] and promote appropriate settings for children’s PA by encouraging parents to use the most appropriate spaces [48]. Lack of opportunities and transport might be more important to address with parents in rural areas [49].

### Improving parental support of their child’s PA

Parental support has been found to be associated with child PA, and children whose parents provide support and encouragement are more likely to participate in PA and have higher overall levels of PA [33,50-53]. The value of parental support within the 6 to 8 years age range was highlighted, with parents reporting that their child may feel unsure about participating in an activity without their presence. This suggests that interventions to increase children’s PA may need also to address approaches to improving children’s confidence and autonomy.

Many parents talked about their child’s intrinsic motivation towards a particular activity, where this was the principle driver for them to participate in that activity. Children within this age range are motivated by activities that they find fun and engaging whilst parents may prefer other activities either because they rate the skills more highly or because they fit in with parental time schedules [10]. Parental control and structure is essential for family life and normal development but excessive control undermines intrinsic motivation, reducing feelings of self-determination and impeding an inherent need for autonomy [54,55]. This was reported by parents who had presumed that their child would do a certain PA and found that the child did not engage with the behaviour. Balancing the needs of family members and allowing children choice within the realistic constraints of family life, require problem solving and communication skills that may be part of generic parenting programmes.

Children who are more intrinsically motivated are those who perceive themselves as competent and in control of their successes and failures [56,57]. As children’s perceived physical competence is related to their PA involvement [58,59], helping parents to facilitate their child’s feelings of competence is likely to be important. This might be achieved through helping parents to teach their children basic, age-appropriate physical skills and by facilitating parenting styles that influence competence [60].

### Strengths and limitations

This study provides insights into parents’ assessment and views of their child’s PA and provides information on how they can be used to form the content of a parenting programme to increase PA in children. We recruited parents from areas varying in their socio-economic characteristics and interviewed a sufficient number of parents to reach data saturation. However, a number of limitations should be acknowledged including the relatively small sample size, the recruitment of parents in Bristol, UK only, and that our sample included very few fathers. The data relied on parents’ perceptions of their child’s physical activity and may not be an accurate representation of reality (we had no way of knowing how active the children really were).

## Conclusions

This study suggests that parents may not identify a need for their child to increase their PA based on their judgement of their child’s current PA levels. In addition, parents most commonly reported that environmental barriers and time and energy constraints prevented their child from participating in PA. Although these barriers should be considered and addressed within a parenting intervention to increase child PA, other factors, such as parental support for children’s motivation, competence and enjoyment of PA may play a more tangible role in behaviour change. Strategies and practices that enhance these factors may in turn influence long term PA behaviour and could be included in PA parenting interventions.

## Abbreviations

PA: Physical activity; SV: Screen-viewing; SES: Social economic status.

## Competing interests

We have no conflicts of interest to declare.

## Authors’ contributions

The study was designed by RJ, SJS, PJL, KRF, SSB and KMT. The interviews were collected and analysed by GFB and JKG under the supervision of RJ and KMT. The first draft of the paper was written by GFB and all authors provided critical revisions for intellectual content. All authors have approved the final manuscript.

## Pre-publication history

The pre-publication history for this paper can be accessed here:

http://www.biomedcentral.com/1471-2431/12/180/prepub
